# Supplementary material for: Locus coeruleus integrity and the effect of atomoxetine on response inhibition in Parkinson’s disease
Source: Brain. 2021 Mar 30;144(8):2513–26. doi: 10.1093/brain/awab142 (PMC7611672; doi:10.1093/brain/awab142)
Supplement: awab142_Supplementary_Data [file awab142_Supplementary_Data.pdf]

Supplementary material

Locus coeruleus integrity and the effect of atomoxetine on response inhibition in Parkinson's disease

1. Locus coeruleus imaging

i) Study specific atlas comparison Page 2

ii) Left versus right locus coeruleus comparison Page 3

iii) Locus coeruleus 25% probability mask Page 4

2. Behavioural results

i) Mood and behaviour questionnaires Page 5

ii) Motor symptom laterality Page 7

iii) Within session physiological effects Page 8

iv) Within session VAS scores Page 10

3. Ex-Gaussian race model of response inhibition

i) Full list of priors Page 12

ii) Posterior predictive checks Page 13

iii) Group-level means of attentional failure parameters Page 16

iv) Selection of predictors of SSRT Page 17

v) Robustness of linear mixed model results Page 18

vi) Effect of alternative contrast-ratio on drug × CR interaction Page 20

vii) Hemisphere effects on drug × locus coeruleus CNR interaction Page 20

viii) Drug × locus coeruleus CNR interaction using 25% mask Page 20

ix) Control region analysis Page 21

4. Additional stop-signal task details

i) Check list for reporting stop-signal studies Page 23

4. References Page 28

Comparison between study-specific and validated locus coeruleus atlases

We compared the study-specific atlas to a validated locus coeruleus atlas described in.[1] The voxel-wise probability distributions were highly correlated between the validated, published atlas and study-specific atlas (5% version: Pearson's r=0.99; 25% version: Pearson's r=0.97). The morphology of the contour was almost identical as shown in the figure below. The Dice Similarity Coefficients between the validated and study-specific atlases were 0.94 and 0.93 for the 5% and 25% probability versions.

Supplementary Figure 1

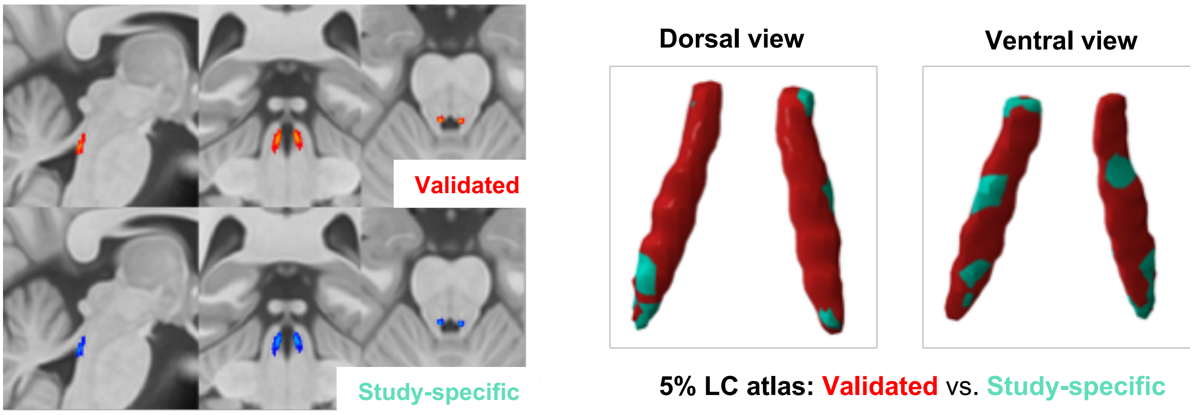

Visual comparisons between validated 7T locus coeruleus atlas and the study-specific atlas generated.

Left versus right locus coeruleus comparison

To explore possible lateralisation effects in the locus coeruleus CNR signal, we performed the same analyses that we applied to the combined CNR values in the main manuscript, but now adding side (i.e., left or right) as an additional within subjects variable. This comparison is shown in Supplementary Figure 2.

When comparing across the whole structure, similar to the main analysis, the groups were not significantly different (main effect of group: $F_{(1,41)} = 1.93$, $p = .172$; BF = 0.65); additionally, there was no main effect of side ($F_{(1,41)} = 0.18$, $p = .676$; BF = 0.23), or group by side interaction ($F_{(1,41)} = 0.73$, $p = .397$; BF = 0.38).

Comparing across the rostral, middle and caudal subdivisions, similar to the main analysis, there was a main effect of subdivision ($F_{(1.34,\ 54.91)} = 65.47$, $p <.001$; BF = 3.21×10^{24}) and there was a significant group by subdivision interaction ($F_{(1.34,\ 54.91)} = 7.97$, $p = .003$; BF = 2787.93). We did not find a main effect of side ($F_{(1,\ 41)} = 0.18$, $p = .676$; BF = 0.14) or a group by side interaction ($F_{(1,\ 41)} = 0.73$, $p = .397$; BF = 0.23). Although there was a significant subdivision by side interaction ($F_{(1.86,\ 76.18)} = 19.95$, $p <.001$; BF = 79.33), crucially, this did not affect the group difference, as evidenced by a non-significant group by subdivision by side interaction ($F_{(1.86,\ 76.18)} = 1.28$, $p = .283$; BF = 0.20). Together, this analysis confirms that the group effects did not change when incorporating locus coeruleus side, arguing against asymmetric degeneration in the patient group.

Supplementary Figure 2

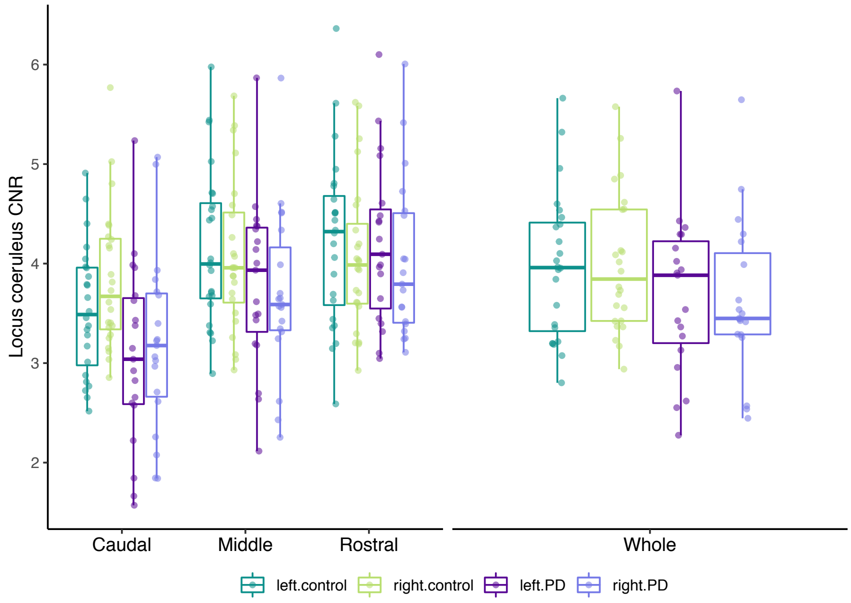

Locus coeruleus 25% probability mask

To verify our results using a more conservative definition of the locus coeruleus, we ran the group comparisons using a 25% probability mask. Supplementary Figure 3 shows comparisons of locus coeruleus CNR between the patients and controls using the 25% mask. As detailed below, the results using the 25% mask were qualitatively identical to the results using the 5% mask reported in the manuscript.

When comparing across the whole structure, the groups were not significantly different ($t_{(36.30)}$ = 1.92, p = .063; BF = 1.33). Comparing across the rostral, middle and caudal subdivisions, there was a main effect of subdivision ($F_{(1.31, 53.52)}$ = 7.48, p = .005; BF = 2.21). This was driven by CNR in the caudal portion being significantly lower than both the middle ($t_{(82)}$ = 3.10, p =.008) and rostral ($t_{(82)}$ = 3.58, p =.002) portions. There was a significant group by subdivision interaction ($F_{(1.31, 53.52)}$ = 14.21, p < 001; BF = 2527.77). This reflected significantly lower CNR values in the caudal portion for patients relative to controls ($t_{(51.6)}$ = 3.40, p = .001), whereas the groups did not differ for the middle ($t_{(51.6)}$ = 1.80, p = .078) or rostral ($t_{(51.6)}$ = 0.301, p = .762) portions of the locus coeruleus.

Supplementary Figure 3

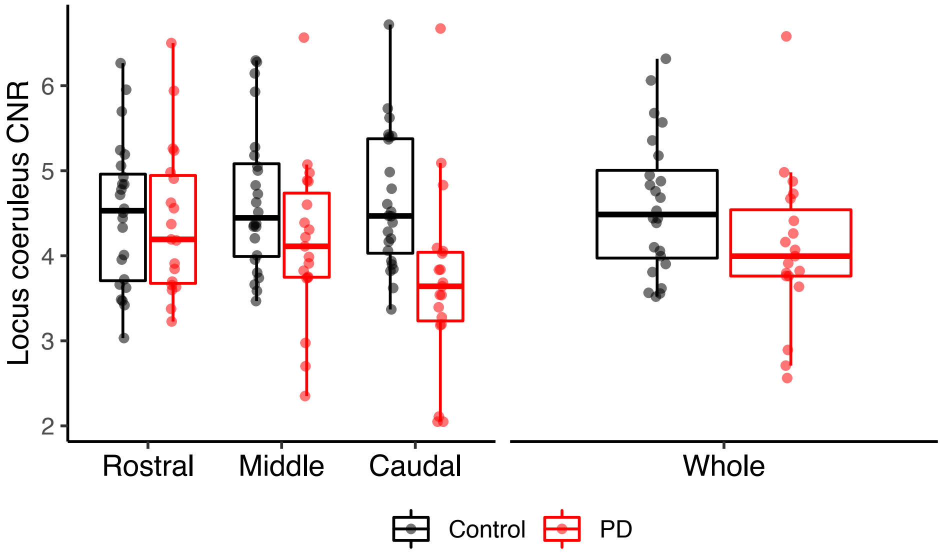

Mood and behaviour questionnaires

Individuals with Parkinson's disease completed self-rated questionnaires to assess mood and behavioural symptoms. These included assessment of anxiety and depression (Hospital Anxiety and Depression scale; HADS)[2], impulsivity (Barratt Impulsiveness Scale; BIS-11[3]; Conners' Adult ADHD Rating Scale; CAARS)[4], apathy (Apathy Scale[5]; Motivation and Energy Inventory; MEI)[6] and REM sleep behaviour disorder (REM sleep behaviour disorder screening questionnaire; RBDSQ.[7] Controls also completed all of these self-rated questionnaires, apart from the RBDSQ. Informant-rated questionnaires were collected from a relative or friend of the patients. These included informant versions of the CAARS and AS, and a general mood and behaviour symptom inventory (Cambridge Behavioural Inventory Revised; CBI-R.[8]

Supplementary Table 1

**Measure**		**PD**	**Controls**	**BF_{10}**	***p***
Apathy Scale	Total Score (self-rated)	12.68 (5.77)	10.58 (5.09)	0.58	.212
	Total Score (informant-rated)	13.13 (5.59)			
BIS	Total Score	56.45 (10.34)	56.15 (9.67)	0.3	.924
	Attention	14.16 (4.3)	14.23 (3.72)	0.3	.953
	Motor	20.08 (2.65)	20.85 (3.38)	0.39	.398
	Non-planning	22.21 (5.4)	21.08 (4.44)	0.38	.459
HADS	Anxiety	4.53 (3.2)	4.31 (3.53)	0.3	.83
	Depression	3.95 (2.68)	2.88 (2.76)	0.58	.202
MEI	Total Score	98.05 (21.3)	108.96 (16.71)	1.29	.073
	Mental	44.11 (8.97)	47.35 (8.09)	0.57	.22
	Physical	23.95 (6.95)	29.35 (5.91)	**6.18**	**.01**
	Social	30 (7.34)	32.27 (5.31)	0.53	.261
CAARS (self-rated)	Inattention / Memory Problems	5.42 (3.58)	4.19 (3.06)	0.55	.235
	Hyperactivity / Restlessness	3.11 (2.71)	2.77 (2.05)	0.33	.652
	Impulsivity / Emotional Lability	1.84 (1.5)	2.77 (2.05)	0.9	.087
	Problems with Self-Concept	2.26 (2.33)	3.88 (4.12)	0.77	.102
	ADHD Index	6.79 (4.26)	8.38 (4.51)	0.53	.233
CAARS (observer-rated)	Inattention / Memory Problems	4.24 (2.51)			
	Hyperactivity / Restlessness	1.58 (1.92)			

	Impulsivity / Emotional Lability	1.21 (1.23)
	Problems with Self-Concept	2.32 (2.11)
	ADHD Index	4.47 (3.42)
RBDSQ		4.58 (3.45)
	Total Score	15.13 (13.6)
	Abnormal Behaviour	0.84 (1.12)
	Beliefs	0.37 (1.21)
	Eating Habits	0.95 (1.58)
	Everyday Skills	1.16 (2.41)
CBI	Memory and Orientation	4.66 (3.9)
	Mood	1.26 (2.1)
	Motivation	2.26 (3.35)
	Stereotypic and Motor Behaviours	0.79 (1.4)
	Self Care	0.42 (0.84)
	Sleep	2.42 (2.17)

Note: Data are presented as mean (SD). Group comparisons were performed with independent samples t-tests. BF_{10}, default Bayes Factor for the alternative hypothesis versus the null hypothesis; *p*, two-tailed *p*-values, uncorrected for multiple comparisons.

Supplementary Figure 4

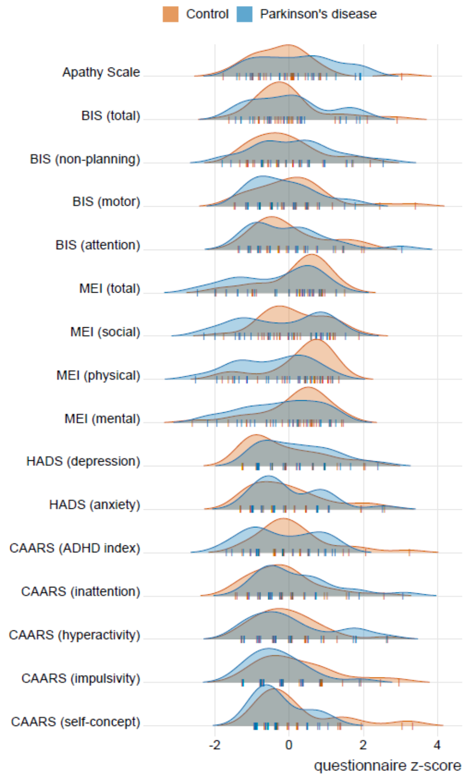

Density plots of questionnaire outcomes for the patient (blue) and control (orange) groups. Each questionnaire outcome was z-scored to facilitate visual comparisons of different questionnaires (note that group comparisons for a given questionnaire outcome are unaffected by this transformation). Tick marks reflect individual data points.

Motor symptom laterality

Using the items from the MDS-UPDRS-III that have separate scores for the left and right side of the body, we calculated a motor asymmetry index (MAI). MAI was calculated as:

$$\frac{\text{Right side symptoms} - \text{Left side symptoms}}{\text{Right side symptoms} + \text{Left side symptoms}}$$

Results from a one-sample t-test showed that the distribution of people with left vs. right dominant motor symptoms did not differ from zero (M = -0.01, SD = 0.43; $t_{(18)}$ = -0.15, p = .883; *BF* = 0.24), and we conclude that there is no motor symptom laterality bias in our cohort.

Supplementary Figure 5

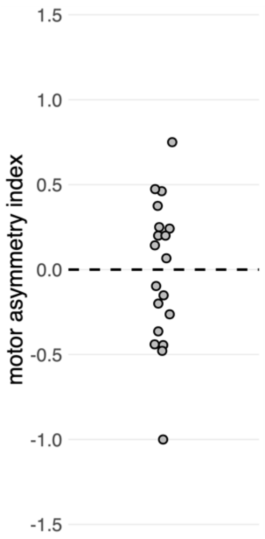

Plot showing individuals with a dominance of left-sided symptoms (negative MAI) versus a dominance of right-sided symptoms (positive MAI). MAI = motor asymmetry index.

Within session physiological effects

As described below, there was evidence of increased pulse rates under atomoxetine, when assessed in the upright (but not the supine) position. Systolic and diastolic blood pressure was also increased under atomoxetine for supine (but not upright) measures. There was evidence for a time effect on supine blood pressure measures, where they were raised at completion of testing, relative to the measures on arrival and two-hours post tablet administration. Mean values and ranges for blood pressure and pulse rates are shown in Supplementary Table 2.

Pulse rates

Supine/Lying down pulse rates did not change significantly under atomoxetine vs. placebo, as evidenced by a lack of main effect ($F_{(1, 89)} = 5.57$, $p = .020$; BF = 1.91), and did not vary across the three time points (i.e., arrival, two-hours post tablet, on completion of testing; $F_{(2, 89)} = 3.00$, $p = .055$; BF = 0.90). Upright pulse rates were increased under atomoxetine, showing a significant main effect ($F_{(1, 88.03)} = 20.99$, $p < .001$; BF = 629.81), and a significant interaction between drug status and time point ($F_{(2, 88.03)} = 6.43$, $p = .002$; BF = 13.75) driven by higher pulse rates under atomoxetine at two hours post administration ($t_{(88)} = 10.93$, $p < .001$, BF = 4.56) and on completion of testing ($t_{(88)} = 10.39$, $p < .001$, BF = 6.57).

Blood pressure

Supine systolic and diastolic blood pressure was raised under atomoxetine, as evidenced by significant main effects (systolic: $F_{(1, 89)} = 9.86$, $p = .002$; BF = 13.39; diastolic: $F_{(1, 89)} = 16.21$, $p < .001$; BF = 163.03). Upright systolic and diastolic blood pressure did not show an overall change under atomoxetine, as evidenced by the lack of main effects (systolic: $F_{(1, 88)} = 2.41$, $p = .124$; BF = 0.57; diastolic: $F_{(1, 88)} = 0.00$, $p = .972$; BF = 0.19). However, there was a main effect of time point (systolic: $F_{(1, 88)} = 5.86$, $p = .004$; BF = 8.47; diastolic: $F_{(1, 88)} = 5.98$, $p = .004$; BF = 8.24), driven by increased blood pressure on completion of testing, compared to arrival and two hours post (systolic, arrival vs. completion: $t_{(88)} = 9.77$, $p = .011$, BF = 1.94; systolic, two hours post vs. completion: $t_{(88)} = 9.79$, $p = .010$, BF = 3.52; diastolic, arrival vs. completion: $t_{(88)} = 4.87$, $p = .012$, BF = 2.30; diastolic, two hours post vs. completion: $t_{(88)} = 5.60$, $p = .006$, BF = 3.99).

Supplementary Table 2

**Measure**			**Placebo**	**Atomoxetine**
Pulse rates	Lying	Arrival	70.00 (33.5 – 55.5; 8.85)	70.58 (56 – 91; 10.27)
		2-hours	69.95 (55 – 86; 10.37)	74.89 (58 – 95; 11.25)
		Completion	66.95 (50 – 85; 8.20)	70.21 (50 – 93; 11.06)
	Upright	Arrival	75.63 (49 – 100; 12.64)	74.95 (56 – 110; 14.30)
		2-hours	70.00 (54 – 86; 8.88)	80.95 (60 – 106; 13.91)
		Completion	68.33 (49 – 80; 7.88)	79.95 (57 – 116; 15.39)
Systolic blood pressure	Lying	Arrival	127.68 (84 – 151; 16.67)	133.26 (109 – 175; 17.24)
		2-hours	125.32 (95 – 156; 15.03)	135.11 (114 – 169; 15.35)
		Completion	131.21 (116 – 155; 12.54)	136.58 (83 – 186; 24.03)
	Upright	Arrival	124.37 (80 – 165; 21.78)	130.26 (90 – 166; 21.20)
		2-hours	123.21 (101 – 145; 12.47)	131.37 (93 – 167; 18.68)
		Completion	139.11 (118 – 176 14.60)	136.11 (97 – 183; 20.99)
Diastolic blood pressure	Lying	Arrival	70.89 (50 – 84; 8.61)	74.00 (55 – 85; 7.46)
		2-hours	68.37 (54 – 80; 6.68)	74.53 (58 – 94; 9.04)
		Completion	72.42 (59 – 87; 7.07)	77.21 (63 – 98; 9.93)
	Upright	Arrival	72.89 (43 – 82; 9.71)	73.32 (56 – 86; 8.09)
		2-hours	71.26 (51 – 91; 10.78)	73.47 (52 – 93; 9.82)
		Completion	79.44 (61 – 93; 8.15)	76.68 (61 – 92; 9.08)

Note: Data are presented as mean (range; SD).

Within session subjective effects

Although the visual analogue scale (VAS) is a continuous measure, participants often respond at either end of the scale, leading to bi- or even tri-modal distributions (Supplementary Figure 6). Such dynamics are not well captured by conventional analyses (e.g., linear regression) that assume multivariate normality. To address this issue, we analysed the VAS data using a Bayesian ordered beta regression model.[9] The strength of this model is that it simultaneously estimates the probability of responses at the scale's lower and upper bounds as well as continuously distributed responses in between the bounds.

Supplementary Figure 6

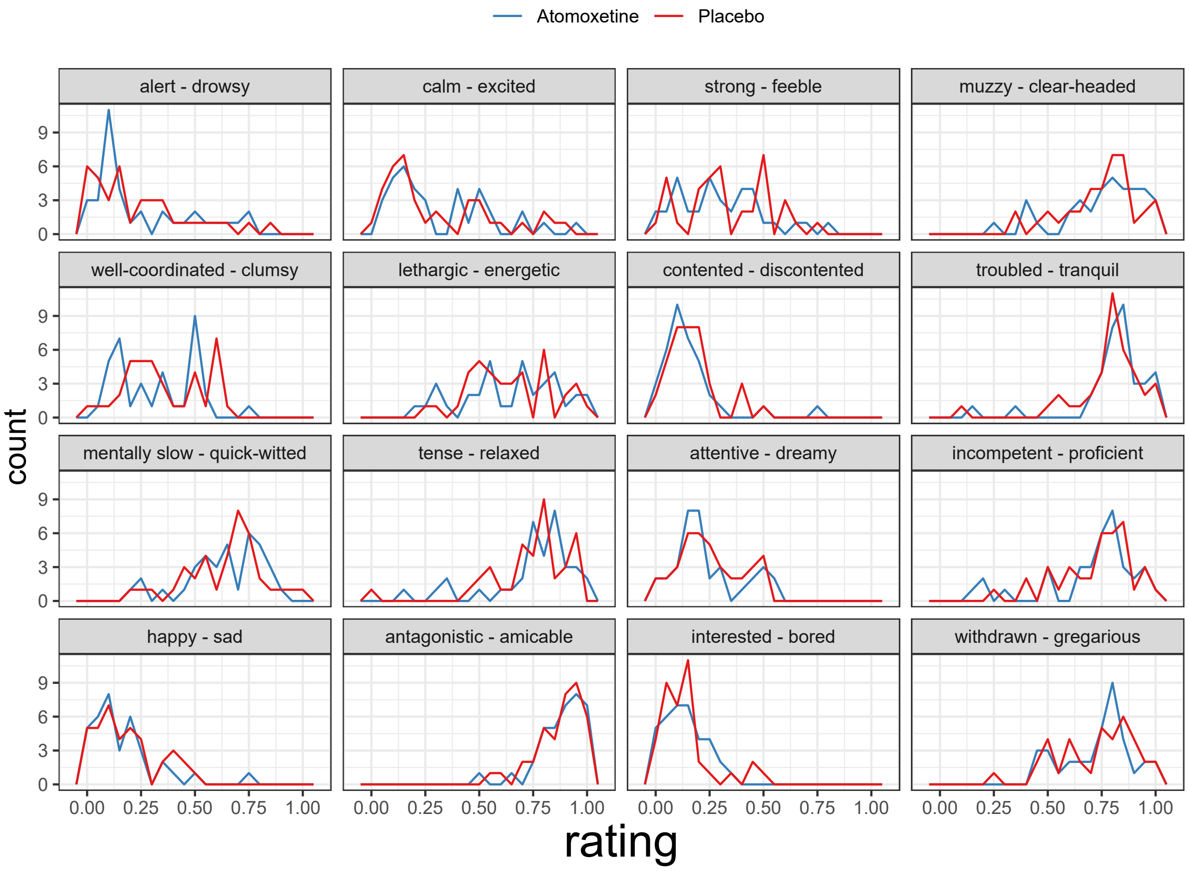

Frequency polygons of VAS ratings in the PD group. Each panel represents one VAS item, as described by the panel titles. The first term of each panel title corresponds to the left extreme of that VAS item (i.e. rating = 0), whereas the second term corresponds to the right extreme (i.e. rating = 1).

We modelled drug (atomoxetine vs. placebo), time point (2 hours post administration vs. baseline), the drug × time interaction, and session (first vs. second visit) as categorical predictors of the VAS response (i.e., fixed effects), and we allowed the intercept to vary by VAS item and by participant (i.e., random effects). Following Kubinec (2020), we assigned a weakly informative normal prior on the regression coefficients: $\beta \sim N(0,5)$. For posterior

inference, we set a region of practical equivalence (ROPE) at $\pm 0.1 \times SD_{VAS} = \pm 0.019$, corresponding to a negligible effect size.[10,11]

There were no main effects of drug or time point on VAS response, as the posterior distributions of these coefficients were largely contained by the ROPE (Supplementary Figure 3; drug: β = -0.02, 95% HDI [-0.06, 0.03], proportion in ROPE = 46.20%; time point: β = 0.02, 95% HDI [-0.03, 0.06], proportion in ROPE = 49.69%). Although the posterior estimate of the drug × time point interaction effect was greater than the upper bound of the ROPE, we failed to reject the null as a relatively large proportion of the posterior distribution was contained by the ROPE (interaction: β = 0.03, 95% HDI [-0.01, 0.08], proportion in ROPE = 24.01%). Taken together, these results suggest that atomoxetine did not induce a significant change in subjective states, as measured by the VAS.

Supplementary Figure 7

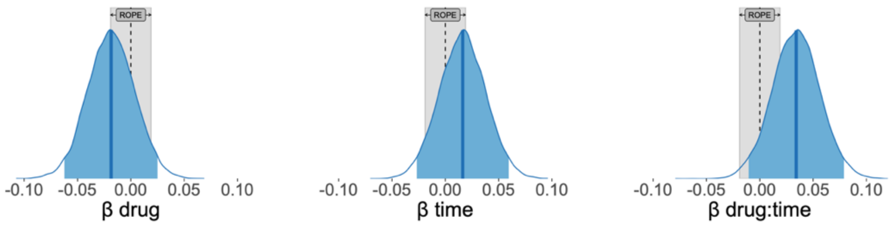

Posterior distributions of predictors of VAS responses. For each panel, the dark blue vertical line represents the median – that is, the posterior estimate of the regression coefficient; the blue shaded area represents the 95% highest density interval of the posterior distribution; and the blue density trace represents the full posterior distribution. The grey area represents a region of practical equivalence (ROPE), corresponding to a negligible effect size of ±0.1.

Full list of priors

Supplementary Table 3 lists the prior distributions assigned to the group-level mean μ and standard deviation σ of each parameter of the ex-Gaussian race model of response inhibition. These priors are identical to those used by the model developers,[12] except for higher prior mean values for the group-level means of $\mu_{go-match}$ and $\mu_{go-mismatch}$ (both 1.5s instead of 0.5s) as well as μ_{stop} (1s instead of 0.5s), to account for slower RT in older age and neurodegenerative disease.

Supplementary Table 3

Parameter	Group-level prior distributions		
Mean of Gaussian component of matching go process ($\mu_{go-match}$)	$\mu_{\mu_{go-match}}$<br>$\sigma_{\mu_{go-match}}$	~<br>~	$N_+(1.5, 1)$<br>$\exp(1)$
Mean of Gaussian component of mismatching go process ($\mu_{go-mismatch}$)	$\mu_{\mu_{go-mismatch}}$<br>$\sigma_{\mu_{go-mismatch}}$	~<br>~	$N_+(1.5, 1)$<br>$\exp(1)$
Mean of Gaussian component of stop process (μ_{stop})	$\mu_{\mu_{stop}}$<br>$\sigma_{\mu_{stop}}$	~<br>~	$N_+(1, 1)$<br>$\exp(1)$
Standard deviation of Gaussian component of matching go process ($\sigma_{go-match}$)	$\mu_{\sigma_{go-match}}$<br>$\sigma_{\sigma_{go-match}}$	~<br>~	$N_+(0.2, 1)$<br>$\exp(1)$
Standard deviation of Gaussian component of mismatching go process ($\sigma_{go-mismatch}$)	$\mu_{\sigma_{go-mismatch}}$<br>$\sigma_{\sigma_{go-mismatch}}$	~<br>~	$N_+(0.2, 1)$<br>$\exp(1)$
Standard deviation of Gaussian component of stop process (σ_{stop})	$\mu_{\sigma_{stop}}$<br>$\sigma_{\sigma_{stop}}$	~<br>~	$N_+(0.2, 1)$<br>$\exp(1)$
Mean of exponential component of matching go process ($\tau_{go-match}$)	$\mu_{\tau_{go-match}}$<br>$\sigma_{\tau_{go-match}}$	~<br>~	$N_+(0.2, 1)$<br>$\exp(1)$
Mean of exponential component of mismatching go process ($\tau_{go-mismatch}$)	$\mu_{\tau_{go-mismatch}}$<br>$\sigma_{\tau_{go-mismatch}}$	~<br>~	$N_+(0.2, 1)$<br>$\exp(1)$
Mean of exponential component of stop process (τ_{stop})	$\mu_{\tau_{stop}}$<br>$\sigma_{\tau_{stop}}$	~<br>~	$N_+(0.2, 1)$<br>$\exp(1)$
Probit-transformed trigger failure probability [$\phi(P_{TF})$]	$\mu_{\phi(P_{TF})}$<br>$\sigma_{\phi(P_{TF})}$	~<br>~	$N(\phi(0.1), 1)$<br>$\exp(1)$
Probit-transformed go failure probability [$\phi(P_{GF})$]	$\mu_{\phi(P_{GF})}$<br>$\sigma_{\phi(P_{GF})}$	~<br>~	$N(\phi(0.1), 1)$<br>$\exp(1)$

Where $N(\mu, \sigma)$ denotes a normal distribution with mean μ and standard deviation σ; $N_+(\mu, \sigma)$ denotes a normal distribution truncated to allow only positive values; and $\exp(\lambda)$ denotes an exponential distribution with rate parameter λ. All parameters except trigger and go failure are on the scale of seconds.

Goodness of fit: Posterior predictive checks

Supplementary Figure 8

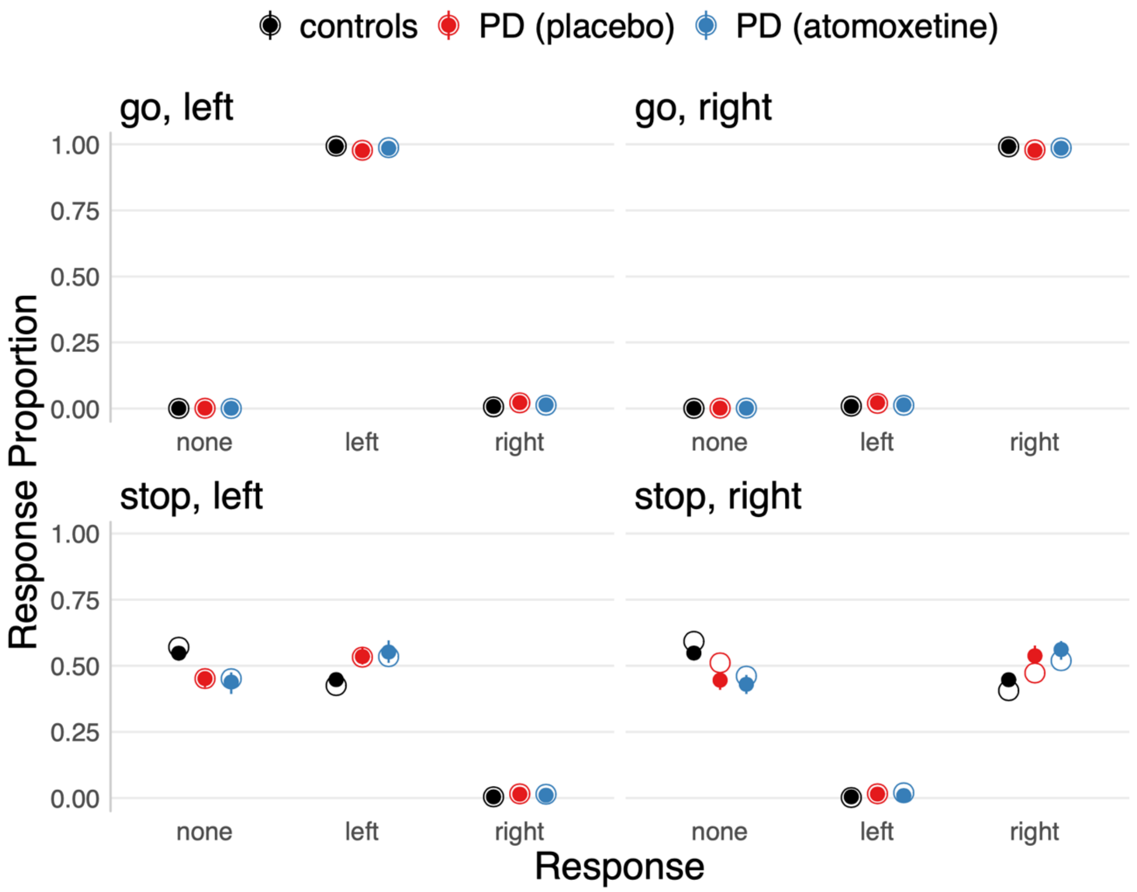

Posterior predictive check of response proportions: Comparing experimentally acquired data (hollow dots) to simulated results from the final model fit (solid dots). Each figure panel represents one combination of trial type (go or stop) and stimulus (left or right). Within each panel, the group-level median response proportions are plotted separately for each response (no response, left, right) and group (controls, PD placebo, PD atomoxetine). The model predictions are illustrated as the median (solid dot) and 95% quantile interval (error bars) of 100 simulated participants, randomly drawn from the joint posterior distribution.

Supplementary Figure 9

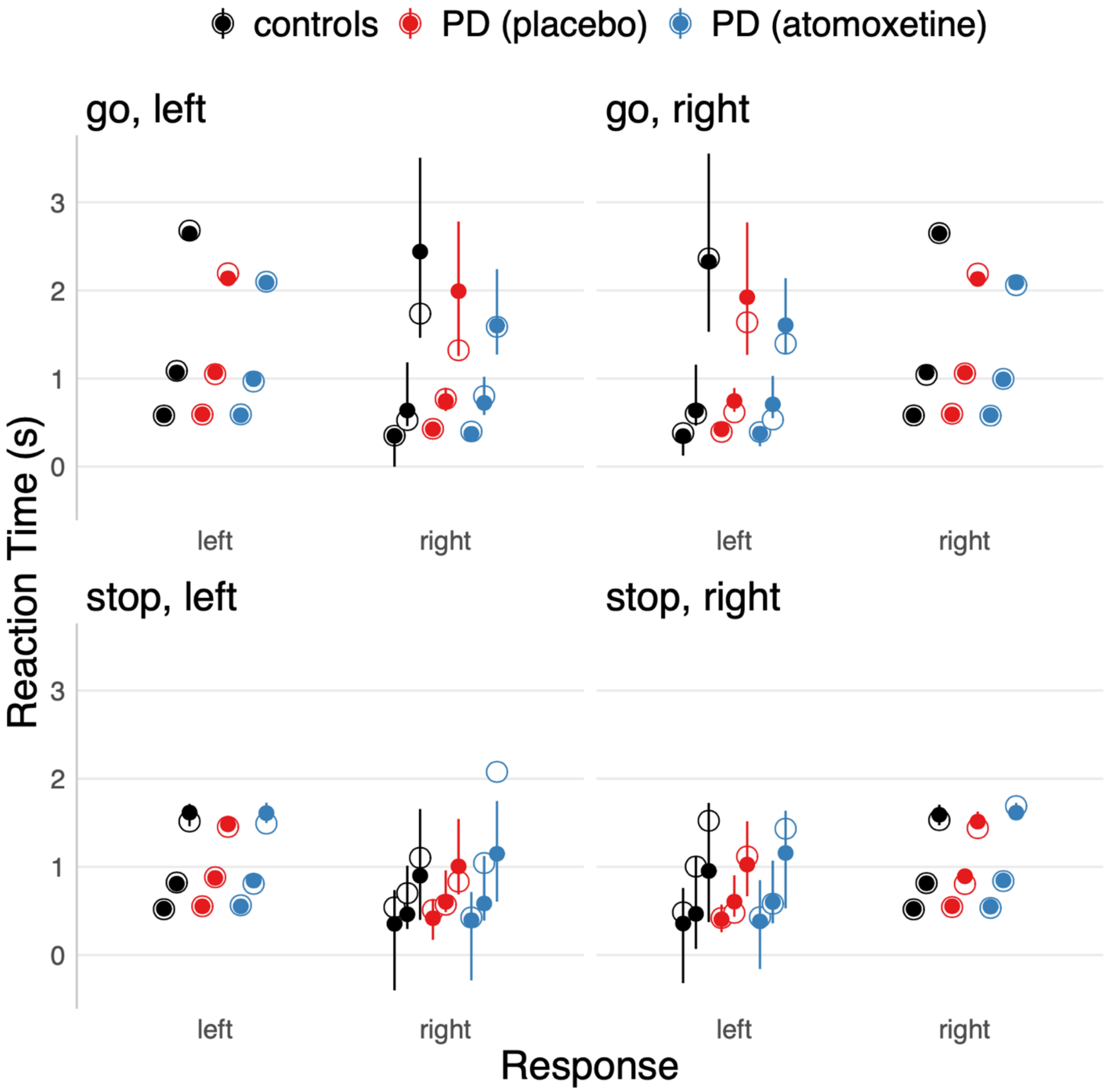

Posterior predictive check of reaction times: Comparing experimentally acquired data (hollow dots) to simulated results from the final model fit (solid dots). Each figure panel represents one combination of trial type (go or stop) and stimulus (left or right). Within each panel, the lower, middle and upper set of dots represent the 10th, 50th, and 90th percentiles of the reaction time distributions, respectively. The model predictions are illustrated as the median (solid dot) and 95% quantile interval (error bars) of 100 simulated participants, randomly drawn from the joint posterior distribution.

Supplementary Figure 10

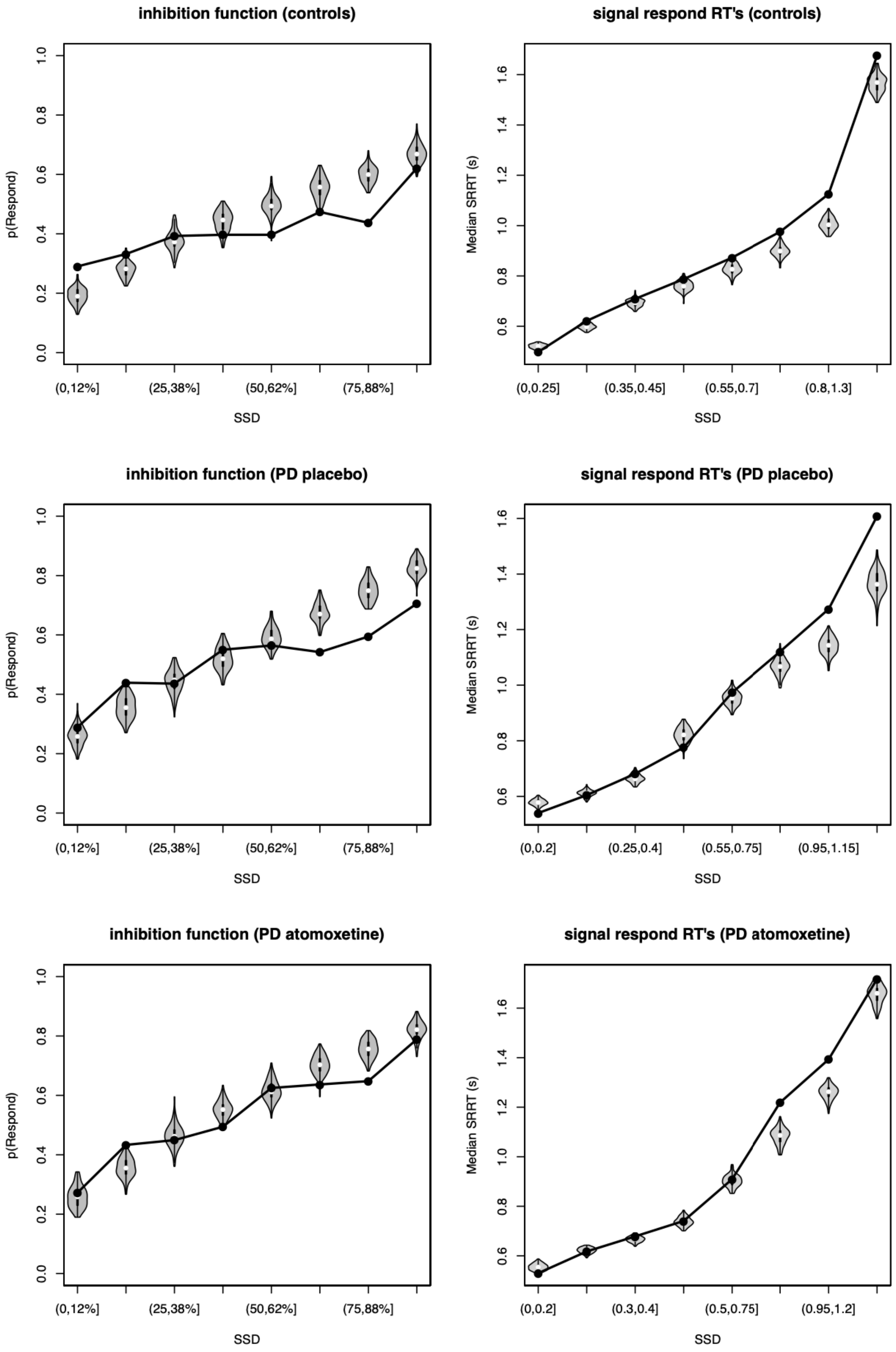

Posterior predictive checks for stopping performance: Comparing experimentally acquired data (black dots and lines) to simulated results from the final model fit (grey violin plots). The panels in the left column illustrate the mean probability of responding (i.e., mean probability of stop failure) as a function of the stop signal delay (SSD). The panels in the right column illustrate the median reaction time for stop trials (i.e., median signal respond RT), as a function of the SSD. Given the high variability of the range of SSD's across participants, the SSD was binned by percentile ranges (left column) or ranges of raw SSD's (right column). The violin plots illustrate the uncertainty of model predictions, obtained from randomly simulating 100 participants from the model's joint posterior distribution. The white dots within the violin plots represent the medians of the model predictions.

Group-level means of attentional failure parameters

Supplementary Figure 11

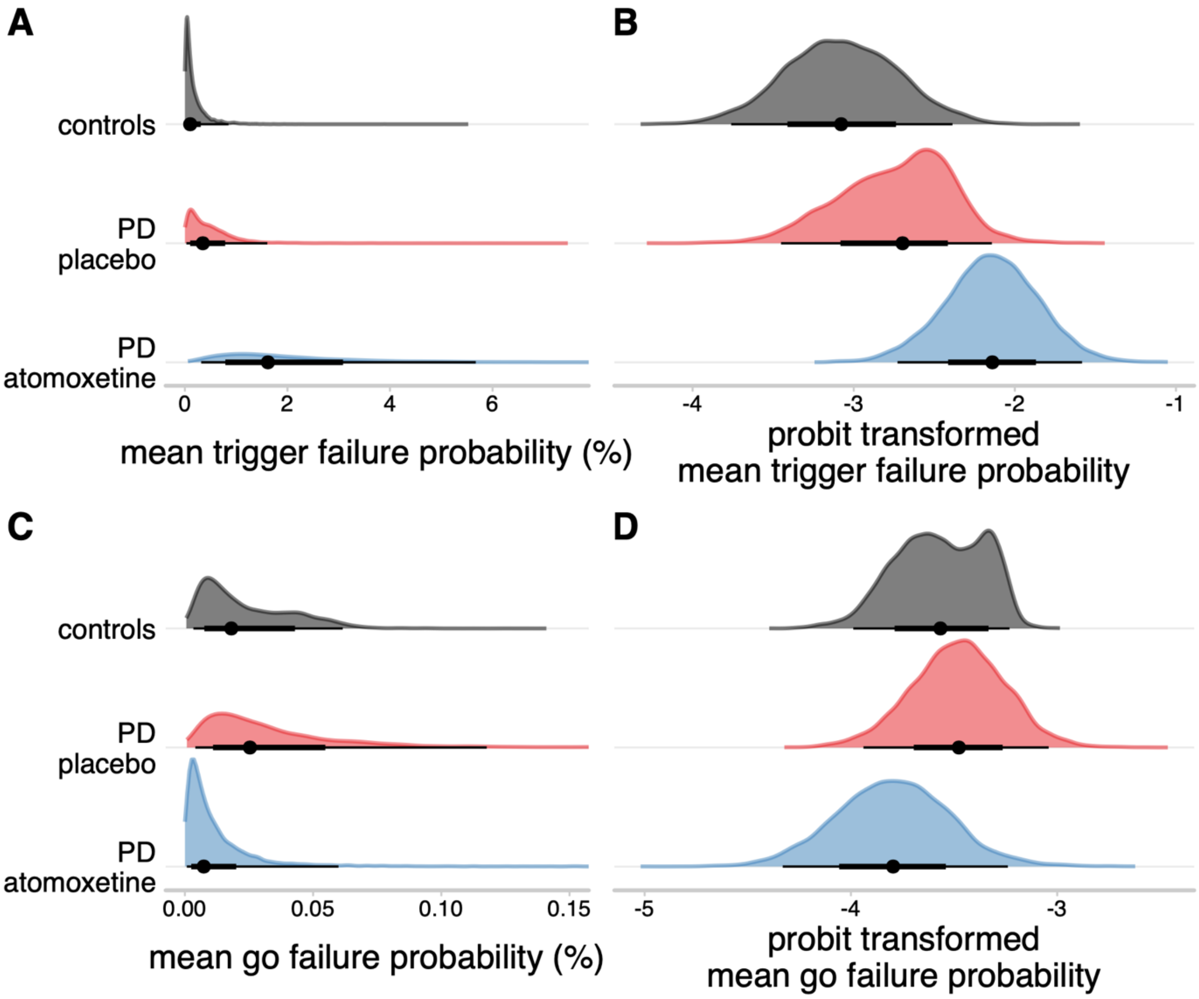

Posterior distributions of the group-level mean of trigger failure probability (A) and go failure probability (C). These distributions were projected from the probability scale to the real line (-∞, ∞) using the probit function, yielding (approximately) normally distributed posterior distributions for mean trigger failure (B) and mean go failure (D). For the probit transformed mean trigger failure probability, there were no meaningful differences between the control group (median = -3.08, 95% QI: [-3.76, -2.39]) and the Parkinson's disease group on placebo (median = -2.70, 95% QI: [-3.45, -2.14]; Δgroup median = -0.34, 95% QI: [-1.22, 0.51]). There was also no clear difference in trigger failure between the Parkinson's disease group on atomoxetine (median = -2.14, 95% QI: [-2.73, -1.58]) and on placebo (Δdrug median = 0.57, 95% QI: [-0.24, 1.52]). For the probit transformed mean go failure probability, there were similarly no differences between the control group (median = -3.57, 95% QI: [-3.99, -3.23]) and the Parkinson's disease group on placebo (median = -3.48, 95% QI: [-3.94, -3.04]; Δgroup median = -0.08, 95% QI: [-0.65, 0.41]). The Parkinson's disease group on atomoxetine (median = -3.80, 95% QI: [-4.33, -3.24]) was again not reliably different from the placebo session (Δdrug median = -0.32, 95% QI: [-0.97, 0.36]).

Selection of predictors of SSRT

Supplementary Table 4. Backward elimination of fixed effects in the linear mixed model predicting SSRT.

	**Model selection step**				
**Predictors**	1	2	3	4	5
Drug	-0.05	-0.05	-0.05	-0.05	-0.05
LC CNR	0.13	0.09	0.14	0.11	0.11
**Drug × LC CNR**	0.27**	0.27**	0.27**	0.27**	**0.27****
**Session**	0.25**	0.25**	0.25**	0.25**	**0.25****
UPDRS III	0.42	0.44	0.35	0.33	-
Ato plasma	-0.16	-0.15	-0.14	-	-
Age	-0.13	-0.15	-	-	-
LEDD	0.08	-	-	-	-
AIC	90.99	89.14	87.48	85.96	86.56
Δ AIC	5.03	3.18	1.53	0	0.60
BIC	108.41	104.98	101.74	98.63	97.64
Δ BIC	10.76	7.33	4.10	0.98	0

Note. Values for predictors are standardised regression coefficients (ß). $^{**}p < .01$. Drug: atomoxetine vs. placebo condition; LC CNR: Locus Coeruleus Contrast to Noise Ratio; Session: first vs. second session; UPDRS III: Unified Parkinson's Disease Rating Scale, motor examination; Ato plasma: atomoxetine plasma concentration; LEDD: Levodopa Equivalent Daily Dose; AIC: Akaike Information Criterion; BIC: Bayesian Information Criterion; Δ AIC / BIC: difference in AIC / BIC with respect to the lowest AIC / BIC value. All models included a random effect of participants on the intercept.

Supplementary Table 5. Inclusion probabilities and Bayes factors for the inclusion of fixed effects in the linear mixed model predicting SSRT.

**Predictors**	P(incl)	P(incl|data)	P(excl|data)	$BF_{inclusion}$
Drug	0.40	0.12	0.31	0.38
LC CNR	0.40	0.18	0.25	0.71
**Drug × LC CNR**	0.20	0.57	0.05	**11.83**
**Session**	0.50	0.76	0.24	**3.16**
UPDRS III	0.50	0.54	0.46	1.21
Ato plasma	0.50	0.43	0.57	0.78
Age	0.50	0.43	0.57	0.76
LEDD	0.50	0.42	0.58	0.74

Note. P(incl): prior inclusion probability, i.e. the summed prior probability of models that include the predictor. A priori, all possible restrictions of the full model were deemed to be equally likely (i.e., a uniform prior was assigned to the model space). Thus, P(incl) reflects the proportion of alternative models that included the predictor. P(incl|data): posterior inclusion probability, i.e. the summed posterior probability of models that include the predictor. P(excl|data): posterior exclusion probability, i.e. the summed posterior probability of models that exclude the predictor. $BF_{inclusion}$: Inclusion Bayes Factor, i.e. the change from prior to posterior inclusion odds. This indicates how much more likely the data are under models that include the predictor, compared to models that exclude the predictor.[13] This analysis was performed using "matched" models, which means that (i) models were not permitted to include an interaction effect without its constituent main effects, and (ii) inclusion probabilities for an interaction effect were based only on the subset of models that contained (at least) the constituent main effects of the interaction.[14] All models included a random effect of participants on the intercept.

Robustness of linear mixed model results

Several standardised measures have been proposed to detect overly influential data points in the context of linear mixed models.[15] The basic rationale behind these measures is that when individual participants are iteratively removed from the data, the linear mixed models based on these data should produce roughly similar parameter estimates. In other words, if the removal of one specific participant causes a drastic change in the model output, this participant should be considered as overly influential.

Cook's distance[16,17] provides a summary measure of the influence that a given participant exerts on all parameters of the linear mixed model simultaneously. As a rule of thumb, a participant is regarded as too influential if their Cook's distance exceeds 4 divided by the total number of participants.[18] The Cook's distance values in our sample ranged from approximately zero to 0.12, well within the applicable cut-off value of 4/19 = 0.21. See Supplementary Figure 12A.

Since we were specifically interested in the Drug × LC CNR interaction term, we also computed DFBETA,[19] which indicates the influence a given participant exerts on a single parameter within the linear mixed model. The conventional cut-off value for DFBETA is equal to ±2 divided by the square root of the total number of participants.[20] We found that the absolute DFBETA values for the Drug × LC CNR interaction term ranged from 0.02 to 0.39, which is within the applicable cut-off value of 0.49. See Supplementary Figure 12B.

As a final effort to ensure the robustness of our linear mixed model results, we also re-fit the model using a robust estimation method (`robustlmm` package in R[21]), which is less sensitive to outliers in the data than the conventional squared error loss function. The results of this robust analysis were qualitatively identical to the conventional linear mixed model analysis presented in the manuscript. Specifically, there was a significant Drug × LC CNR interaction effect ($\beta = 0.27$, $t = 3.39$, $p = .004$), as well as a significant main effect of Session ($\beta = 0.25$, $t = 3.24$, $p = .006$), but no significant main effects of Drug ($\beta = -0.05$, $t = -0.74$, $p = .470$) or LC CNR ($\beta = 0.11$, $t = 0.46$, $p = .654$).

Taken together, multiple diagnostic tools indicated that our sample did not contain any overly influential participants. This suggests that the parameter estimates of our linear mixed model were not particularly susceptible to the influence of any given participant. The lack of influential data can be seen as an endorsement of the quality of the model fit and generalisability of the results.[15]

Supplementary Figure 12

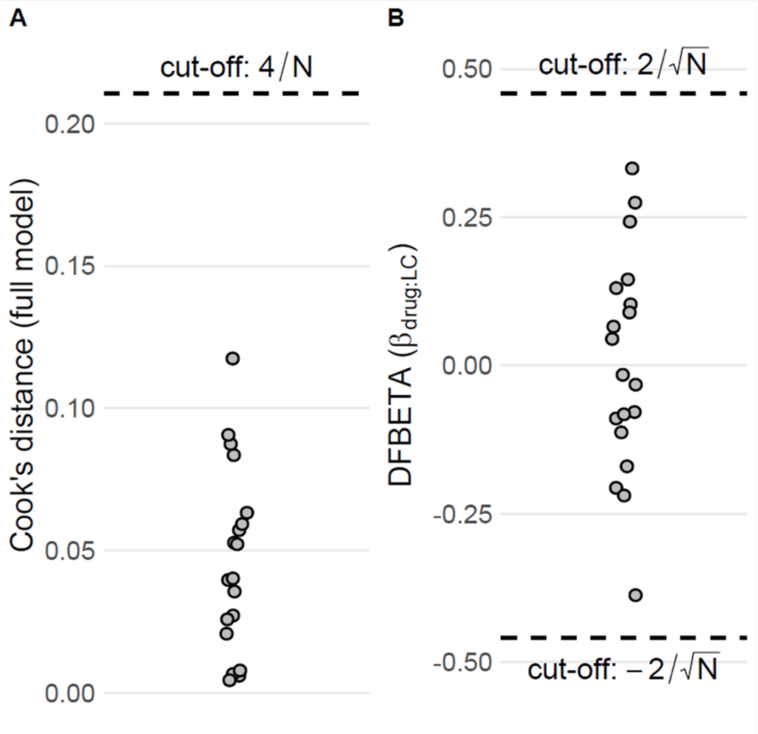

Effect of alternative contrast-ratio on drug × CR interaction

We re-ran the linear mixed model using an alternative calculation of locus coeruleus contrast: CR = (V - $Mean_{REF}$)/($Mean_{REF}$), to confirm that the results were not driven by the CNR estimation method that we chose for the main results. The results of this analysis were qualitatively similar to the original analysis presented in the manuscript. Specifically, we found a significant Drug × LC CR interaction effect (β = 0.21, F = 7.50, p = .016; BF = 2.39), as well as a significant main effect of Session (β = 0.20, F = 7.44, p = .016; BF = 1.57), but no significant main effects of Drug (β = -0.05, F = 0.49, p = .496; BF = 0.40) or LC CR (β = -0.41, F = 4.13, p = .058; BF = 1.81).

Hemisphere effects on drug × locus coeruleus CNR interaction

To explore the possibility of hemisphere-specific effects, we ran the linear mixed model analysis separately for CNR values from the left and right locus coeruleus. As detailed below, these results were qualitatively identical to the original combined analysis, arguing against hemisphere-specific effects.

For the left LC, there was a significant Drug × LC CNR interaction effect (β = 0.25, F = 10.84, p = .005; BF = 4.99), as well as a significant main effect of Session (β = 0.25, F = 11.09, p = .005; BF = 1.61), but no significant main effects of Drug (β = -0.05, F = 0.64, p = .436; BF = 0.37) or LC CNR (β = 0.09, F = 0.16, p = .694; BF = 0.54). For the Right LC, there was a significant Drug × LC CNR interaction effect (β = 0.27, F = 15.55, p = .001; BF = 14.05), as well as a significant main effect of Session (β = 0.25, F = 13.51, p = .002; BF = 2.90), but no significant main effects of Drug (β = -0.05, F = 0.74, p = .404; BF = 0.36) or LC CNR (β = 0.10, F = 0.22, p = .642; BF = 0.55).

Drug × locus coeruleus CNR interaction using 25% mask

We ran the linear mixed model analysis using the CNR values derived from the more conservative 25% probability mask. These results were qualitatively similar to the results obtained using the 5% mask. There was a significant Drug × LC CNR interaction effect (β = 0.28, $F_{(1, 14.43)}$ = 19.14, p < .001; BF = 27.14), as well as a significant main effect of Session (β = 0.25, $F_{(1, 14.39)}$ = 15.13, p = .002; BF = 4.68), but no significant main effects of Drug (β = -0.06, $F_{(1, 14.18)}$ = 0.89, p = .362; BF = 0.37) or LC CNR (β = 0.13, $F_{(1, 17.02)}$ = 0.35, p = .563; BF = 0.56).

Control region analysis

To confirm that the drug × locus coeruleus CNR interaction did not change when considering non-specific brain imaging metrics, we calculated total intracranial volume and substantia nigra CNR, and included them as covariates in the linear mixed model.

Methodological details

To estimate total intracranial volume (ICV), the T1-weighted MP2RAGE images were processed using the FreeSurfer (v6.0.0) recon-all procedure (https://surfer.nmr.mgh.harvard.edu/fswiki/recon-all) and ICV was obtained using "mri_segstats --etiv-only".[22] We also added the high resolution option (https://surfer.nmr.mgh.harvard.edu/fswiki/SubmillimeterRecon) used for recon-all for data with voxel sizes less than 1mm^3.[23]

To obtain substantia nigra CNR, we first created an independent probabilistic substantia nigra ROI using a semi-automated approach in the independent sample of 29 age- and education-matched healthy controls. This procedure is similar to the approach in the main manuscript for the independent LC atlas creation, which was validated in Ye et al.[1] Using those 29 subjects' magnetisation transfer-weighted (MT) scans, we identified a searching area in the midbrain based on the Atlas of the Basal Ganglia and Thalamus (ABGT)[24,25] substantia nigra boundaries. We selected reference regions (left and right) in the midbrain background (*crus cerebri*). For each subject, voxels were selected within the search area that were above the threshold, T:

$$T = AV_{REF} + 5 \times SD_{REF}$$

We then averaged across the subjects' segmentations and thresholded the probabilistic ROI at 5% to obtain a mask. Then, to extract substantia nigra CNR from the study sample, we quantified contrast by establishing the CNR with respect to reference regions in the left and right midbrain.[26] A CNR map was computed voxel-by-voxel on the average MT image for each subject using the signal difference between a given voxel (V) and the mean intensity in the reference region ($Mean_{REF}$) divided by the standard deviation (SD_{REF}) of the reference signals ($CNR = \frac{V - Mean_{REF}}{SD_{REF}}$). CNR values were extracted bilaterally on the CNR map by applying the independent substantia nigra probabilistic mask (5% probability version). For analysis purposes, we combined the left and right substantia nigra values.

Results

When total intracranial volume (ICV) was added as a covariate in the linear mixed model (i.e., `SSRT ~ drug * LC_CNR + ICV + visit + (1 | subject)`), the results were qualitatively identical to the original model that did not include ICV. There was a significant Drug × LC CNR interaction effect (β = 0.27, F = 14.57, p = .002; BF = 11.50), as well as a significant main effect of Session (β = 0.25, F = 13.30, p = .003; BF = 2.75), but no significant main effects of ICV (β = 0.21, F = 0.80, p = .384; BF = 0.81), Drug (β = -0.05, F = 0.71, p = .414; BF = 0.37) or LC CNR (β = 0.15, F = 0.44, p = .518; BF = 0.61).

When substantia nigra CNR was added as a covariate in the linear mixed model (i.e., `SSRT ~ drug * LC_CNR + SN_CNR + visit + (1 | subject)`), the results were qualitatively identical to the original model that did not include substantia nigra CNR. There was a significant Drug × LC CNR interaction effect (β = 0.27, F = 14.54, p = .002; BF = 11.77), as well as a significant main effect of Session (β = 0.25, F = 13.27, p = .003; BF = 2.77), but no significant main effects of substantia nigra CNR (β = -0.06, F = 0.06, p = .807; BF = 0.68), Drug (β = -0.05, F = 0.70, p = .417; BF = 0.36) or LC CNR (β = 0.13, F = 0.31, p = .588; BF = 0.59).

Together, these results point towards a greater specificity in our findings as neither a general measure of brain atrophy nor a neuromelanin-sensitive measure of disease severity further explained variation in SSRT performance, above and beyond the significant interaction we found between LC integrity and atomoxetine.

Additional stop-signal task details

Below we provide details about the task conforming to the "Check list for reporting stop-signal studies" suggested by Verbruggen et al.[27]

Stimuli and materials

Properties of the go stimuli, responses, and their mapping

An overview of the task stimuli is given in the Figure below. All stimuli were presented in the centre of the screen, on a light grey background (RGB code: [195, 195, 195]). The stimuli were pre-loaded as Psychtoolbox textures at the start of the session, to ensure that they could be drawn rapidly to the screen during a trial. For each trial, a left- or right-pointing arrow was presented following a black fixation cross ("+" symbol). For go trials, participants responded to the black stimulus using a two-button response box, where the left button corresponded to a "left" response and the right button corresponded to a "right" response.

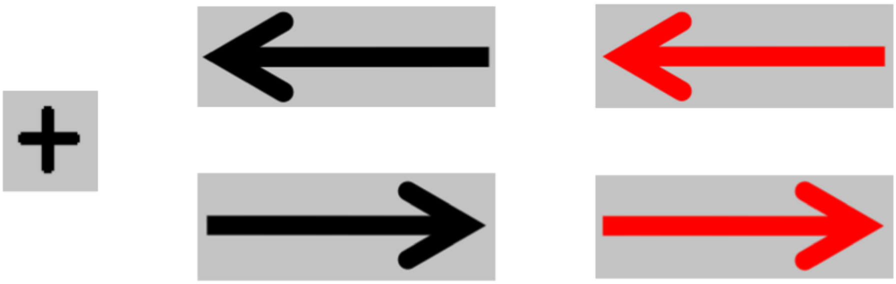

Properties of the stop signal

On stop trials, the left- or right-pointing black arrow was replaced by a corresponding red arrow after the stop signal delay (see Figure above). At the same time, a "beep" sound (1000 Hz pure tone) was played for 100 ms. The loudness of the tone was not systematically controlled; the speaker volume was adjusted as necessary to ensure that the participants could clearly hear the tone, but were not startled by it.

Equipment used for testing

The experiment was run on a Windows 7 laptop, using Matlab R2018B in conjunction with the Psychtoolbox extensions (version 3)[28]. The laptop had a 15-inch screen with a resolution of 1920 × 1080 pixels, and the laptop's built-in speakers were used to play the stop signal tone. The two-button response box was placed directly in front of the laptop on a desk, and was connected to the laptop via a standard USB 2.0 connection.

Procedure

Number of blocks

The task consisted of 1 practice block, followed by 4 test blocks.

Number of go and stop trials per block

The practice block consisted of 20 go trials, 2 no-go trials, and 3 stop trials. Each testing block consisted of 110 go trials, 10 no-go trials, and 20 stop trials.

Trial order randomisation

At the start of each block, the trial sequence was pseudorandomly ordered as follows. The first 20 trials were go trials, used to compute a starting SSD value (see below). The remaining 120 trials (90 go trials, 10 no-go trials, 20 stop trials) were pseudorandomly ordered, with the constraints that there could be no more than 7 consecutive go trials, 2 consecutive no-go trials, and 2 consecutive stop trials. For the practice block, the first 5 trials were go trials, and the remaining 20 trials (15 go trials, 2 no-go trials, 3 stop trials) were pseudorandomly ordered using the same constraints as above.

SSD tracking procedure

For each block, a starting SSD value was computed as the mean RT of the initial go trials (20 trials for testing blocks, 5 trials for practice block) minus 200 ms. In the unlikely event that this initial mean RT was faster than 200 ms, the starting SSD value was set to 200 ms. For the remainder of the block, the SSD was adjusted using a staircase method to target a stop accuracy of 50%. Specifically, the SSD increased by 50 ms following successful inhibition (i.e. response withheld), and decreased by 50 ms following failed inhibition (i.e. response given). The SSD was constrained to range from 50 ms to 1500 ms.

Event timings

- intertrial interval: not applicable.
- fixation interval: At the start of each trial, a fixation cross was presented for 500 ms. The only exception was the first trial of the first testing block, where the fixation cross was presented for 2 s.
- stimulus presentation:
- For go trials, the black arrow was presented on the screen for a maximum of 5 s. If the participant performed a response, the arrow was removed from the screen immediately following button release, and the next trial began.

- For stop trials, the black arrow was presented on the screen for the duration of the SSD, and subsequently the red arrow was presented on the screen for 2 s minus the SSD. That is, the total duration of a stop trial was 2 s. The trial was not terminated in case of a response, including failed stop responses (i.e. RT > SSD) and premature responses (i.e. RT ≤ SSD).
- For no-go trials, the red arrow was immediately presented on the screen, and remained on the screen for 2 s (regardless of whether participants responded or not).
- maximum response latency: The response deadlines corresponded to the maximum duration of stimulus presentations. Thus, the response deadline was 5 s for go trials, and 2 s for stop and no-go trials.
- feedback duration: not applicable.

Summary of instructions and feedback-related information

At the start of the testing session, participants read the following instructions from the screen:

In this experiment you will make button presses as quickly and accurately as you can.
Each time you see a BLACK LEFT-pointing arrow, press the LEFT button as fast as you can.
For a BLACK RIGHT-pointing arrow, press the RIGHT button as fast as you can.

[Please press either the left or right button to continue]

Sometimes, you will see a RED arrow. This indicates you must NOT PRESS any button.
Sometimes, after a black arrow appears you will hear a TONE and the arrow turns RED. This indicates that you have to STOP yourself from pressing a button.

[Please press either the left or right button to continue]

You should always try to respond as QUICKLY AND ACCURATELY AS YOU CAN to each arrow as it appears.
You will not receive error messages if you make mistakes, so just try your best to respond correctly.

[Please press either the left or right button to continue]

TO SUMMARISE:
Please respond using the left and right buttons.
Please try as hard as you can to be fast, accurate, and stop when possible after hearing a tone and seeing the red arrow.
If you have any questions, please ask now.
When you are ready to do some practice trials, press either the left or right button.

The experimenters checked that participants fully understood the task after the practice block, and if necessary the practice block was repeated (although this was rarely the case). Participants

were not given any specific feedback about their task performance, and there was no formal debriefing at the end of the testing session.

Training procedures

There was no specific training besides the practice block.

Analyses

Data pre-processing

Descriptive statistics of task performance (go error rate, stop accuracy rate) were based on the full dataset (i.e. without pre-processing). Prior to model fitting, the following pre-processing steps were performed. First, all no-go trials were removed (40 per participant). Second, any trials with RT's faster than 0.25 s or slower than 4.5 s were removed from the data, as these were considered to be contaminants that were not generated from the ex-Gaussian race process (e.g. accidental button presses or misses, fast guesses)[29]. Third, go trials with RT's more extreme than the mean go RT ±2.5 SD's were removed on a participant-by-participant basis. On average, 6.77 trials (1.32%) were removed per participant (range: 0 – 26 trials, 0% – 5%), not including the removal of no-go trials. We note that preliminary model fitting without the latter two pre-processing steps yielded parameter estimates that were practically identical to those from the final model fits.

SSRT estimation method

The SSRT was inferred using hierarchical Bayesian estimation of a parametric race model of the stop signal task. The model and its fitting procedure are described in detail elsewhere,[12,30] as well as in the Methods section of the current manuscript (section 'Ex-Gaussian race model of response inhibition'). To summarise, the model assumes a race between three processes: a go runner that matches the stimulus, a go runner that mismatches the stimulus, and a stop runner. The finish times of these runners are assumed to follow ex-Gaussian distributions, with mean μ, SD σ, and slow tail τ. The model further assumes that for a given trial, the go and / or stop processes might not have been triggered, such that there was no competitive race between the runners. These attentional failures are accounted for with two additional probabilistic parameters, trigger failure and go failure, which represent the probability that the stop process and go processes were not triggered, respectively. Thus, in total the model features 11 free parameters: The three parameters of the ex-Gaussian distribution for each of the three processes, plus the two attentional failure parameters. The SSRT corresponds to the mean of the ex-Gaussian distribution of the stop process, which is given by $\mu + \tau$.

The model parameters were estimated using Bayesian hierarchical methods, such that parameters for individual participants were considered to be samples from corresponding group-level normal distributions. The priors for the group-level mean and SD of each parameter are given in the Supplementary Material ('Full list of priors'). All model fitting was performed using the Dynamic Models of Choice toolbox.[12]

Statistical analyses

Group-level parameter estimates from the parametric race model were compared by examining the overlap between the 95% quantile intervals (QI) of posterior distributions. Specifically, if there was no overlap between the 95% QI's of two posterior distributions, they were considered to be significantly different from each other. Participant-level parameter estimates from the parametric race model were subjected to both frequentist and Bayes factor (BF) analyses for hypothesis testing. In particular, linear mixed models were used to examine the effects of Drug (placebo vs. atomoxetine), LC CNR, and their interaction on parameter estimates for the participants with Parkinson's disease. For frequentist analyses, p-values for fixed effects were obtained using the Kenward-Roger method, with a significance threshold of p = .05 (two-sided). For Bayesian analyses, inclusion BF's for fixed effects were obtained using Bayesian model averaging to estimate the change from prior to posterior inclusion odds.[13] The BF's were interpreted using Kass and Raftery's[31] classification scheme, such that BF > 3 constitutes "positive evidence".

Descriptive statistics (separately for each group and condition)

	PD (placebo)	PD (atomoxetine)	controls
Basic task parameters			
go omission errors	0.11% (0.30%)	0.09% (0.32%)	0.05% (0.13%)
choice errors on go trials	2.05% (2.54%)	1.33% (1.97%)	0.73% (1.47%)
unsuccessful stop trials	51.60% (14.99%)	54.58% (12.88%)	42.00% (18.40%)
mean stop-signal delay	0.72 s (0.42 s)	0.69 s (0.45 s)	0.81 s (0.46 s)
mean RT of go responses on unsuccessful stop trials	1.06 s (0.38 s)	1.15 s (0.56 s)	1.06 s (0.43 s)
Ex-Gaussian model estimates			
mean go RT (matching go runner)	1.23 s (0.51 s)	1.18 s (0.54 s)	1.36 s (0.70 s)
SSRT	0.50 s (0.07 s)	0.49 s (0.07 s)	0.39 s (0.04 s)

Note. Values are given as mean (standard deviation).

References

1. Ye R, Rua C, O'Callaghan C, et al. An in vivo probabilistic atlas of the human locus coeruleus at ultra-high field. *NeuroImage*. 2021;225:117487. doi:10.1016/j.neuroimage.2020.117487

2. Zigmond AS, Snaith RP. The Hospital Anxiety and Depression Scale. *Acta Psychiatrica Scandinavica*. 1983;67(6):361-370. doi:10.1111/j.1600-0447.1983.tb09716.x

3. Patton JH, Stanford MS, Barratt ES. Factor structure of the barratt impulsiveness scale. *Journal of Clinical Psychology*. 1995;51(6):768-774. doi:10.1002/1097-4679(199511)51:6<768::AID-JCLP2270510607>3.0.CO;2-1

4. Conners CK, Erhardt D, Epstein JN, Parker JDA, Sitarenios G, Sparrow E. Self-ratings of ADHD symptoms in adults I: Factor structure and normative data. *J Atten Disord*. 1999;3(3):141-151. doi:10.1177/108705479900300303

5. Starkstein SE, D P, Mayberg HS, et al. Reliability, validity, and clinical correlates of apathy in Parkinson's disease. *Journal of Neuropsychiatry and Clinical Neurosciences*. Published online 1992:134.

6. Fehnel SE, Bann CM, Hogue SL, Kwong WJ, Mahajan SS. The development and psychometric evaluation of the motivation and energy inventory (MEI). *Qual Life Res*. 2004;13(7):1321-1336. doi:10.1023/B:QURE.0000037502.64077.4d

7. Stiasny-Kolster K, Mayer G, Schäfer S, Möller JC, Heinzel-Gutenbrunner M, Oertel WH. The REM sleep behavior disorder screening questionnaire—A new diagnostic instrument. *Movement Disorders*. 2007;22(16):2386-2393. doi:10.1002/mds.21740

8. Wear HJ, Wedderburn CJ, Mioshi E, et al. The Cambridge Behavioural Inventory revised. *Dementia & Neuropsychologia*. 2008;2(2):102-107. doi:10.1590/S1980-57642009DN20200005

9. Kubinec R. Ordered Beta Regression: A Parsimonious, Well-Fitting Model for Survey Sliders and Visual Analog Scales. *SocArXiv*. Published online March 2, 2020. doi:10.31235/osf.io/2sx6y

10. Cohen J. *Statistical Power Analysis for the Behavioral Sciences.* Routledge; 1988.

11. Kruschke JK. Rejecting or Accepting Parameter Values in Bayesian Estimation. *Advances in Methods and Practices in Psychological Science*. 2018;1(2):270-280. doi:10.1177/2515245918771304

12. Heathcote A, Lin Y-S, Reynolds A, Strickland L, Gretton M, Matzke D. Dynamic models of choice. *Behav Res*. 2019;51(2):961-985. doi:10.3758/s13428-018-1067-y

13. Hinne M, Gronau QF, van den Bergh D, Wagenmakers E-J. A Conceptual Introduction to Bayesian Model Averaging. *Advances in Methods and Practices in Psychological Science*. Published online June 2, 2020:2515245919898657. doi:10.1177/2515245919898657

14. Mathôt S. Bayes like a Baws: Interpreting Bayesian Repeated Measures in JASP // Cogsci. Published May 15, 2017. Accessed July 10, 2020. https://www.cogsci.nl/blog/interpreting-bayesian-repeated-measures-in-jasp

15. Nieuwenhuis R, Te Grotenhuis HF, Pelzer BJ. Influence. ME: tools for detecting influential data in mixed effects models. Published online 2012.

16. Cook RD. Detection of influential observation in linear regression. *Technometrics*. 1977;19(1):15-18.

17. Snijders TAB, Berkhof J. Diagnostic checks for multilevel models. In: *Handbook of Multilevel Analysis.* Springer Science + Business Media; 2008:141-175. doi:10.1007/978-0-387-73186-5_3

18. Van der Meer T, Te Grotenhuis M, Pelzer B. Influential cases in multilevel modeling: a methodological comment. *American Sociological Review*. 2010;75(1):173-178.

19. Fox J, Monette G. *An R and S-Plus Companion to Applied Regression*. Sage; 2002.

20. Belsley DA, Kuh E, Welsch RE. *Regression Diagnostics: Identifying Influential Data and Sources of Collinearity*. Vol 571. John Wiley & Sons; 2005.

21. Koller M. robustlmm: an R package for robust estimation of linear mixed-effects models. *Journal of statistical software*. 2016;75(6):1-24.

22. Malone IB, Leung KK, Clegg S, et al. Accurate automatic estimation of total intracranial volume: A nuisance variable with less nuisance. *NeuroImage*. 2015;104:366-372. doi:10.1016/j.neuroimage.2014.09.034

23. Zaretskaya N, Fischl B, Reuter M, Renvall V, Polimeni JR. Advantages of cortical surface reconstruction using submillimeter 7 T MEMPRAGE. *Neuroimage*. 2018;165:11-26.

24. Keuken MC, Bazin P-L, Crown L, et al. Quantifying inter-individual anatomical variability in the subcortex using 7 T structural MRI. *NeuroImage*. 2014;94:40-46.

25. He X, Chaitanya G, Asma B, et al. Disrupted basal ganglia–thalamocortical loops in focal to bilateral tonic-clonic seizures. *Brain*. 2020;143(1):175-190.

26. Chen X, Huddleston DE, Langley J, et al. Simultaneous imaging of locus coeruleus and substantia nigra with a quantitative neuromelanin MRI approach. *Magnetic Resonance Imaging*. 2014;32(10):1301-1306. doi:10.1016/j.mri.2014.07.003

27. Verbruggen F, Aron AR, Band GP, et al. A consensus guide to capturing the ability to inhibit actions and impulsive behaviors in the stop-signal task. *eLife*. 2019;8:e55-26. doi:10.7554/eLife.46323

28. Kleiner M, Brainard D, Pelli D. What's new in Psychtoolbox-3? Published online 2007.

29. Ratcliff R, Tuerlinckx F. Estimating parameters of the diffusion model: Approaches to dealing with contaminant reaction times and parameter variability. *Psychonomic Bulletin & Review*. 2002;9(3):438-481. doi:10.3758/BF03196302

30. Matzke D, Curley S, Gong CQ, Heathcote A. Inhibiting responses to difficult choices. *Journal of Experimental Psychology: General*. 2019;148(1):124-142. doi:10.1037/xge0000525

31. Kass RE, Raftery AE. Bayes Factors. *Journal of the American Statistical Association*. 1995;90(430):773-795. doi:10.1080/01621459.1995.10476572
